# Carbon black-containing self-healing adhesive hydrogels for endoscopic tattooing

**DOI:** 10.1038/s41598-023-28113-1

**Published:** 2023-02-02

**Authors:** Hyung Jun Kwon, Hyun Ho Shin, Da Han Hyun, Ghilsuk Yoon, Jun Seok Park, Ji Hyun Ryu

**Affiliations:** 1 Department of Surgery, School of Medicine, Kyungpook National University Hospital, Kyungpook National University, Daegu, 41404 South Korea; 2 Department of Chemical Engineering, Wonkwang University, Iksan, Jeonbuk 54538 South Korea; 3 Department of Biomedical Science, School of Medicine, Kyungpook National University, Daegu, 41404 South Korea; 4 Department of Pathology, School of Medicine, Kyungpook National University, Daegu, 41566 South Korea; 5 Department of Carbon Convergence Engineering, Wonkwang University, Iksan, Jeonbuk 54538 South Korea; 6 ICT Fusion Green Energy Research Institute, Wonkwang University, Iksan, Jeonbuk 54538 South Korea

**Keywords:** Gastroenterology, Medical research, Engineering, Materials science

## Abstract

Endoscopic tattooing with India ink is a popular method for identifying colonic lesions during minimally invasive surgery because it is highly challenging to localize lesions during laparoscopy. However, there is a perceived unmet need for the injection of India ink and carbon particle suspension due to various complications and inconstant durability during the perioperative period. In this study, carbon black-containing self-healing adhesive alginate/polyvinyl alcohol composite hydrogels were synthesized as endoscopic tattooing inks. Alginate (Alg) conjugated with phenylboronic acid (PBA) groups in the backbone was crosslinked with polyvinyl alcohol (PVA) because of the dynamic bonds between the phenylboronic acid in alginate and the cis-diol groups of PVA. The carbon black-incorporated Alg-PBA/PVA hydrogels exhibited self-healing and re-shapable properties, indicating that improved intraoperative localization could be achieved. In addition, the adhesive tattooing hydrogels were stably immobilized on the target regions in the intraperitoneal spaces. These carbon black-containing self-healing adhesive hydrogels are expected to be useful in various surgical procedures, including endoscopic tattooing.

## Introduction

Laparoscopic surgery has been widely accepted as a standard treatment for colon cancer because of its minimal invasiveness, similar safety, and identical long-term results to conventional open surgery^1–5^. However, the accomplishment of operational procedures depends mainly on subjective experience. Surgeons with vast experience with laparoscopic colectomy can be impeded by the lack of tactile sensation. As a result, a simple and effective strategy for accurate localization of primary lesions during laparoscopic procedures is essential if they are invisible on the serosal surface. For example, small or flat neoplasms, tumors confined to the mucosa and submucosa, and endoscopically resected polyps require additional surgery.

Recently, various materials have been developed for preoperative localization; methylene blue, indigo carmine, toluidine blue, isosulfan blue, hematoxylin and eosin, indoxin green, and India ink have been extensively investigated as endoscopic inks, but few materials are clinically used in colonoscopy^6–12^. In particular, India ink is highly effective for precise tumor localization^7^. However, 8% (4/50) of visible but inaccurate tattoos and 18% (9/50) of invisible tattoos using India ink are judged at operation, as previously reported^8,9^, and 14% of inaccurate localization using various endoscopic tattooing materials has been reported after endoscopic tattooing^10^. Another approach is the autologous blood tattooing method, which involves injecting the patient’s blood^13^. However, it also has difficulty with the identification of the exact location and oncological safety issues associated with the promotion of cancer growth^14^. Thus, it is highly desirable to develop stably immobilized tattooing materials for colonic lesions.

Suspended carbon particles with purification and sterilization are the most frequently used materials for endoscopic tattooing^15–18^. Although safety issues associated with inflammation and immune responses have been previously reported, the autoclave and filtration of carbon particles significantly prevents inflammations^15–18^. Sterile carbon nanoparticles are highly effective and safe for use as endoscopic markers. However, the above-mentioned problems associated with inaccurate and invisible tattooed lesions remain an unsolved challenge in colonoscopy. These results might be closely related to the lack of high mechanical strength and tissue adhesive properties of the suspended carbon particles. Thus, we hypothesized that an increased elastic modulus and tissue adhesive forces might result in advanced endoscopic tattooing materials, preventing inaccurate and invisible tattooing. This new endoscopic marker would be meaningful because a few material-based approaches have been used to overcome this substantial problem.

In this study, we developed self-healing adhesive carbon-black-incorporated alginate-phenylboronic acid/polyvinyl alcohol (CB/Alg-PBA/PVA) hydrogels as endoscopic tattooing materials. These CB/Alg-PBA/PVA hydrogels showed rapid gelling, self-healing, and highly stretchable properties with remarkable mechanical properties. In addition, these hydrogels exhibit excellent adhesiveness to porcine intestinal tissue and can be effectively utilized as an endoscopic marker. One of the simple methods to prepare tissue adhesives is the conjugation of adhesive functional groups (i.e., NHS esters, cyanoacrylate, aldehydes, catechol, aryl azide, and isocyanate) into the polymer backbones^19^. For instance, catechol-functionalized polymers (i.e., chitosan-catechol^20–22^, hyaluronic acid-catechol^23,24^, and alginate-catechol^25^) exhibit significant enhancements of tissue adhesions compared to unmodified polymers. PBA conjugation also provides adhesive properties by boronate ester formation with glycoproteins, peptidoglycan, and polysaccharides^26–32^. Furthermore, CB-containing hydrogels are long-lasting under physiological conditions compared with hydrogels without CB. As previously reported, self-healable Alg-PBA-based hydrogels, including Alg-PBA and Alg-PBA/PVA hydrogels, have been developed by dynamic linkages between boronic acid and diols^31–36^. However, the stability of hydrogels formed by PBA-diol interactions shows significant dependences on the pKa of PBA indicating that the hydrogels are unstable at the pH below the pKa (~ 8.5) of PBA^36–39^. Thus, these self-healing adhesive CB/Alg-PBA/PVA hydrogels are not limited to endoscopic markers and can be extended to implant materials and adhesive hydrogels.

## Experimental details

### Materials

Alginate sodium salt from brown algae (Alg), 3-aminophenylboronic acid monohydrate (PBA), n-hydroxysuccinimide (NHS), and polyvinyl alcohol (PVA; MW = 85–124 kDa) were purchased from Sigma-Aldrich (Milwaukee, WI, USA). 1-Ethyl-3-(3-dimethyl aminopropyl)carbodiimide (EDC) was purchased from TCI-SU (Tokyo, Japan). Carbon black (CB, 30 nm) was purchased from the Graphene Supermarket (Ronkonkoma, NY, USA). All other chemicals were of analytical grade.

### Synthesis and characterizations of phenylboronic acid-conjugated alginate (Alg-PBA)

Alg-PBA was synthesized by conjugating 3-aminophenylboronic acid groups to the alginate backbone, as previously described^31,32^. Briefly, alginate (1 g) was dissolved in distilled deionized water (DDW, 100 mL). EDC (1.09 g) was dissolved in DDW (30 mL) and added slowly to the alginate solution. After 30 min, PBA (778 mg) and NHS (653 mg) were added to the EDC-activated alginate solution. The reaction time was 12 h, and the pH of the reaction solution was adjusted to 5. The product was dialyzed using a membrane (MWCO: 3.5 kDa, SpectraPor, USA) against 10 mM NaCl solution for two days and DDW for one day. The final purified product was lyophilized. The synthesis of Alg-PBA was confirmed by using either ^1^H NMR (Bruker Avance III, 500 MHz) or UV–Vis spectrophotometer (UV-1900i, Shimadzu, Japan). The degree of PBA conjugation onto the alginate backbone was determined using a UV–Vis spectrophotometer by comparing the absorbance of Alg-PBA at 295 nm and the standard curves of PBA monomers^31^. The calculated degree of PBA conjugation is 9.1%.

### Preparation of carbon black-containing Alg-PBA/polyvinyl alcohol (CB/Alg-PBA/PVA) hydrogels

The CB/Alg-PBA/PVA hydrogels were prepared by homogeneously mixing the CB-containing Alg-PBA solution with the PVA solution. In brief, Alg-PBA (2 wt%) was dissolved in a PBS solution (pH 7.4), and then CB (0.5, 1, 2 mg) was added to the Alg-PBA solution. PVA (10 wt%) was added to the CB/Alg-PBA solution. CB/Alg-PBA/PVA hydrogels were formed within 1 min and stabilized for 10 min. The final concentrations of the Alg-PBA and PVA were fixed at 1 and 5 wt%, respectively. The CB concentration was varied from 0.5 to 2 mg/mL.

### Rheological analysis

Rheological studies of the CB/Alg-PBA/PVA hydrogels were performed using a rotational rheometer (Kinexus Lab +, Netzsch, Germany) with a 20 mm parallel plate. For the frequency sweep measurements, the frequency sweep was varied from 0.1 and 10 Hz at 21 points. For step-strain measurements, the strain was subsequently changed to 0.5, 100, 0.5, 100 and 0.5% for 900 s. (180 s each). The elastic modulus (G′) and viscous modulus (G′′) of the hydrogels were monitored using rheological measurements. All measurements were performed in triplicate.

### In vitro stability test

The in vitro stability of the CB/Alg-PBA/PVA hydrogels was determined by measuring the remaining weight at predetermined time intervals. Briefly, the hydrogels (0.5 mL) were prepared in 2 mL tubes, and PBS solution (1 mL, pH 7.4) was added to the hydrogels. The hydrogels in the PBS solution were incubated at 37 °C. The weights of the remaining hydrogels were measured after removing the supernatants at predetermined time intervals. All measurements were performed in triplicate.

### In vitro tissue adhesion test

The in vitro tissue adhesion properties of the CB/Alg-PBA/PVA hydrogels were evaluated using a universal testing machine (UTM, Instron 5583, Instron, USA) with 50 N load cells. Fresh porcine intestine (Bucknam Butcher’s shop, South Korea) samples were cut into 1 × 1 cm^2^ pieces with a rectangular shape for tissue adhesion tests. The intestinal tissue samples were attached to the edge of polyethylene terephthalic acid film (1 × 5 cm^2^) adherends. Two adherends were overlapped by 1 × 1 cm^2^, and then the hydrogels were applied between the intestinal tissue samples. After 1 min of stabilization, the tensile strength was measured by pulling the probe. All measurements were performed in triplicate.

### Animal experiments

Animal experiments were performed at the Animal Research Laboratory of Kyungpook National University. Hydrogel implantation models were used to evaluate the adhesion and immobilization behaviors of the CB-containing hydrogels. The experiment was conducted with a total of four rats (normal Sprague–Dawley male rats, age 4–8 weeks, with a body weight of 200–250 g) on the 3, 7, 14 and 21 days. All procedures were performed by the same surgical team. The anesthesia was performed with Forane inhalation anesthesia and the surgical gloves were cleaned with ethanol to remove powder particles before starting the surgical procedure. An abdominal skin was shaved and disinfected with Betadine. After implantation of hydrogels onto the abdominal wall, the abdominal skin was performed in four places to prevent leakage only by using the 3–0 Vicryl suture. After all the processes, the rats were sterilized with Betadine and moved to a warm and stable place to recover. After 3, 7, 14 and 21 days of surgery, aspiration anesthesia of Forane was performed according to the recommended amount, hair removal was performed and disinfected with Betadine. An abdominal median incision of the existing incision was performed by using a scalpel. The degree of inflammation and the location of the incised ink was inspected. Ink attachment tissue was fixed with 10% neutral buffer formalin for 24 h and stained with hematoxylin and eosin (H/E).

### Ethics approval

This study is reported in accordance with ARRIVE guidelines. Animal experiments and utilization of normal SD rats were approved by the Animal Review Committee of Kyungpook National University (KNU_2022-0037). All procedures were performed according to the ethical protocols of Kyungpook National University and the National Institutes of Health’s Guide.

## Results and discussion

### Preparation and characterization of CB/Alg-PBA/PVA hydrogels

**Figure 1:**
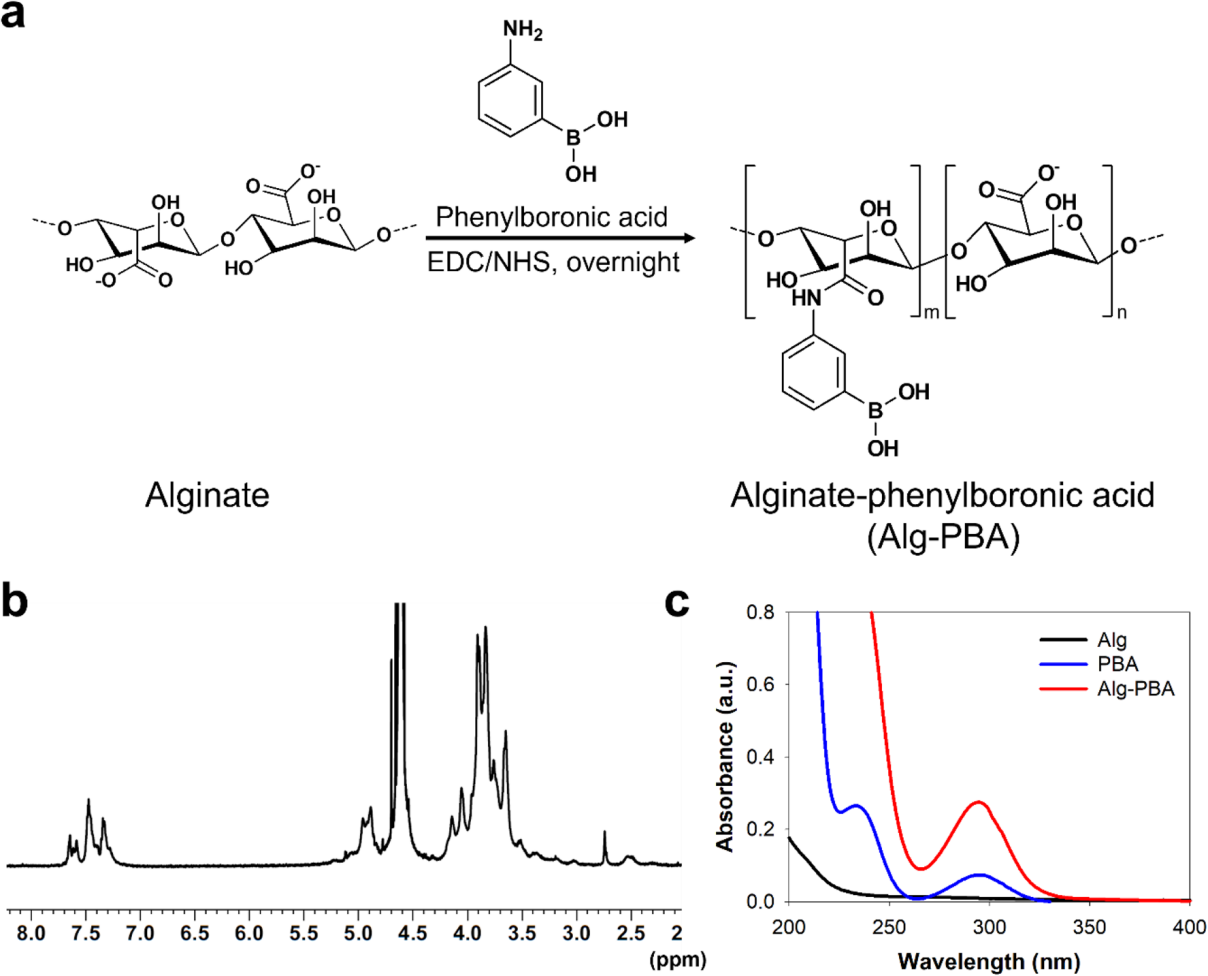
(**a**) Synthesis and chemical structures of phenylboronic acid-conjugated alginate (Alg-PBA), (**b**) ^1^H NMR spectrum of Alg-PBA. **c**) UV–Vis spectra of alginate (black), PBA monomer (blue), and Alg-PBA (red).

To prepare carbon particle-containing self-healing adhesive endoscopic inks, Alg-PBA was synthesized via standard carbodiimide chemistry by forming an amine bond between an amine group in 3-aminophenylboronic acid and carboxylic acid groups in alginate backbones (Fig. 1a). The conjugation of multiple PBA groups onto the alginate backbone was confirmed by ^1^H NMR and UV–Vis spectroscopy. As shown in Fig. 1b, the phenyl group protons of PBA at 7.3 to 7.7 ppm were found in the ^1^H NMR spectra of Alg-PBA. In addition, the synthesized Alg-PBA (red line) showed a peak at 295 nm due to the absorbance of PBA molecules (blue line) in the UV–Vis spectra (Fig. 1c). The degree of PBA substitution in Alg-PBA was 9.1%, as calculated using a UV–Vis spectrophotometer. We used carbon black as suspended carbon particles and polyvinyl alcohol (PVA) as multiple cis-diol sources to form the hydrogels with enhanced long-term stability.

**Figure 2:**
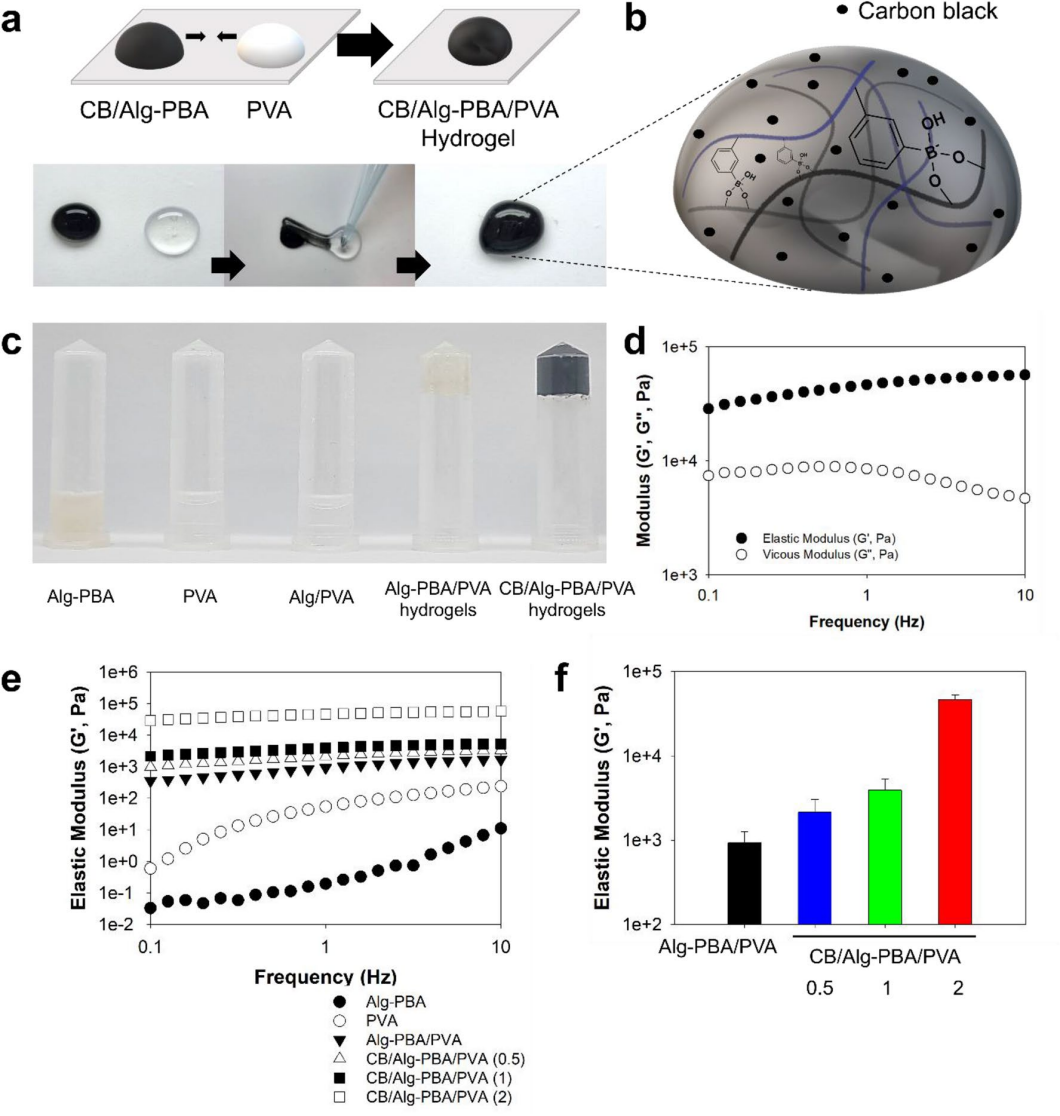
Preparation and characterizations of carbon black-incorporated alginate-phenylboronic acid/polyvinyl alcohol composite hydrogels (CB/Alg-PBA/PVA hydrogels). (**a**) Schematic representative (top) and photos (bottom) of preparations of CB/Alg-PBA/PVA hydrogels. (**b**) Illustration of proposed structures of hydrogels. (**c**) Photographic images of Alg-PBA, PVA, Alg/PVA, Alg-PBA/PVA hydrogels, and CB/Alg-PBA/PVA hydrogels. (**d**) Frequency sweep measurements of CB/Alg-PBA/PVA hydrogels. (**e**) Elastic modulus (G′) changes of samples as a function of frequency. (**f**) Elastic modulus values of CB/Alg-PBA/PVA hydrogels as a function of CB concentration.

To fabricate carbon black-containing alginate/polyvinyl alcohol (CB/Alg-PBA/PVA) composite hydrogels, CB/Alg-PBA and PVA were separately dissolved in PBS solutions of pH 7.4, homogeneously mixed for 1 min, and stabilized for 30 min. After mixing CB/Alg-PBA and PVA, the solution immediately solidified to form hydrogels (Fig. 2a). The gelation of the mixture solution of CB/Alg-PVA and PVA occurred within 2 min (Fig. S1). Figure 2b shows the proposed structures of the CB/Alg-PBA/PVA hydrogel network. The PBA groups in the Alg-PBA were bound to the diol groups of PVA resulting in the formation of 3-dimensional networks and CB was incorporated into the hydrogels. Photographic images of the inversion tube tests of the Alg-PBA/PVA and CB/Alg-PBA/PVA hydrogels provide visual evidence of hydrogel formation (Fig. 2c). When the test tube containing the samples was inverted, no flow along the wall was detected in either the Alg-PBA/PVA or CB/Alg-PBA/PVA hydrogels. In fact, Alg-PBA alone was crosslinked to form hydrogels by phenylboronic acid-cis-diol interactions as previously reported^31,32^. However, Alg-PBA with the low degree of PBA substitution showed no gelation behavior^31,32^. Rheological analysis clearly showed the gelation behavior of the CB/Alg-PBA/PVA hydrogels. For the optimization of CB/Alg-PBA/PVA hydrogels, we measured elastic modulus (G′) values of hydrogels as a function of concentration. As shown in Fig. S2a, the G′ values of Alg-PBA/PVA hydrogels without the CB were increased as a function of PVA concentrations from 1 to 10 wt% at the Alg-PBA concentration of 1 wt%. The G′ values of Alg-PBA/PVA hydrogels with 10 wt% PVA were 2.7 ± 0.6 kPa that are higher than those of hydrogels with 1 wt% PVA (30.2 ± 2.4 Pa), 3 wt% PVA (242.0 ± 16.3 Pa), and 5 wt% PVA (927.5 ± 124.3 Pa). In addition, similar behaviors were observed with different Alg-PBA concentrations (0.5 to 2 wt%) at the PVA concentration of 5 wt% (Fig. S2b). However, the G′ values of CB/Alg-PBA/PVA hydrogels were similar after the addition of 2 mg CB (Fig. S2c). Thus, the final concentrations of the Alg-PBA and PVA were fixed at 1 and 5 wt%, respectively. The CB/Alg-PBA/PVA hydrogels with 2 mg CB showed elastic modulus (G′) values higher than the viscous modulus (G′′) values at an overall frequency between 0.1 and 10 Hz (Fig. 2d). In addition, the elastic modulus values of CB/Alg-PBA/PVA hydrogels significantly increased to 46.4 ± 4.9 kPa, far higher than those of Alg-PBA (0.2 ± 0.2 Pa), PVA (10.5 ± 0.7 Pa), and Alg-PBA/PVA (927.5 ± 261.6 Pa) at a frequency of 1 Hz (Fig. 2e,f). It is noteworthy that incorporating CB into the hydrogel networks could significantly affect both the significant color changes to black and the increased moduli of elasticity. Therefore, these data suggest that CB/Alg-PBA/PVA hydrogels could be used as endoscopic inks with a high elastic modulus.

### Self-healing property of CB/Alg-PBA/PVA hydrogels

**Figure 3:**
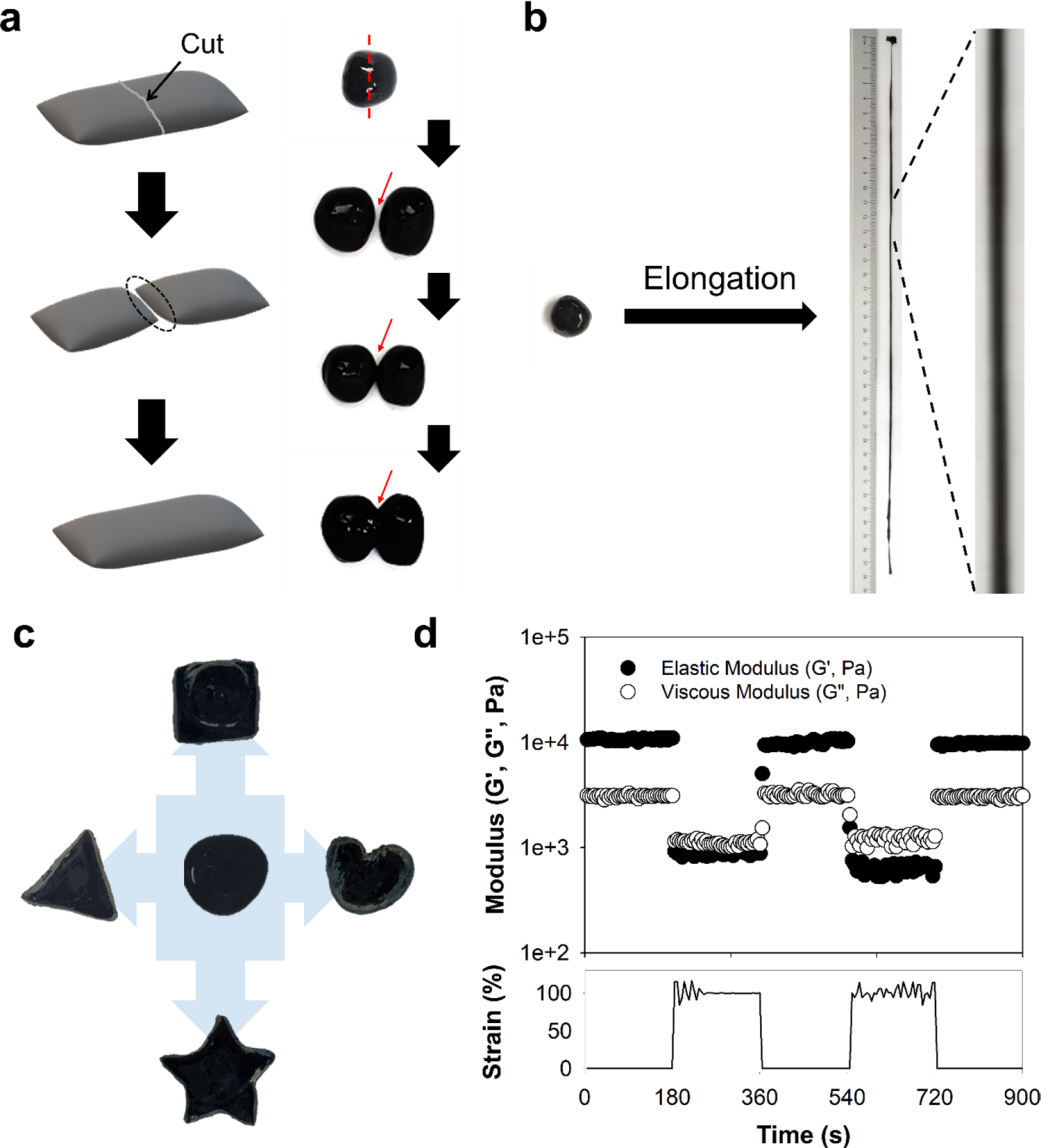
(**a–c**) Photographic images of CB/Alg-PBA/PVA hydrogels containing 2 mg CB to show (**a**) self-healing, (**b**) extensible, and (**c**) re-shapeable properties. (**d**) Step-strain measurements of CB/Alg-PBA/PVA hydrogels.

The dynamic linkages of phenylboronic acid and cis-diols extend the remarkable hydrogel properties, including self-healing, extensibility, and re-shapeability. As shown in Fig. 3a, hydrogels were cut using a blade. Immediately after cutting, the hydrogels instantaneously reattached and completely recovered within 3 min. In addition, the hydrogels were highly extensible (Fig. 3b), and the morphologies readily changed with various shapes, including circle, rectangle, triangle, and star shapes (Fig. 3c). The reorganization of the dynamic linkages of PBA and cis-diols significantly contributed to the rapid self-healing, extensible, and reshaping characteristics^31^. Step-strain measurements were performed using a rotational rheometer (Fig. 3d) to confirm the self-healing behavior of the hydrogels. When a 0.5% strain was applied, the elastic modulus values (G′) of the hydrogels were 10.6 kPa. After applying a 100% strain, these values were slightly reduced to 880.6 Pa. These values were recovered by ~ 93% (9.9 kPa) after applying 0.5% strain. After another step, the elastic modulus values greater than 90% (9.7 kPa) were recovered. This slight reduction might be due to the short recovery time required to form hydrogel networks by reversible PBA and cis-diol interactions.

### Tissue adhesiveness and stability of CB/Alg-PBA/PVA hydrogels

**Figure 4:**
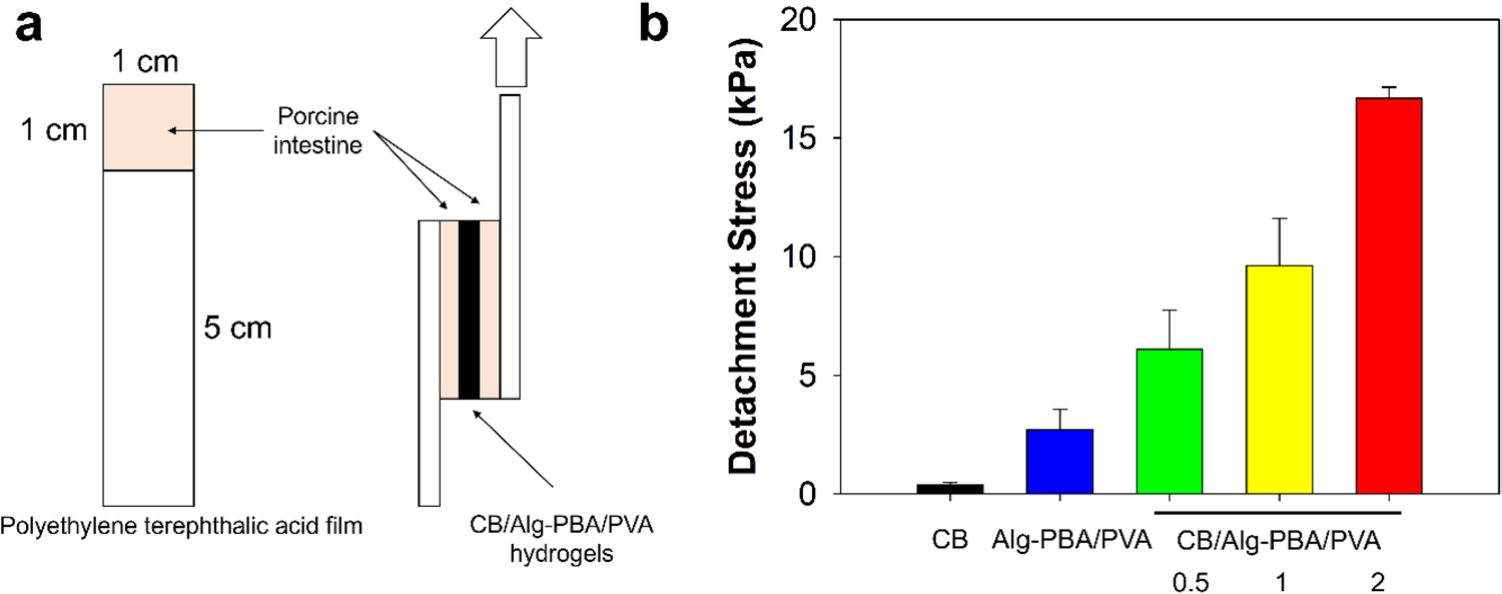
(**a**) Schematic illustrations of the measurements of tissue adhesive property. (**b**) Detachment stress of CB, Alg-PBA/PVA, and CB/Alg-PBA/PVA hydrogels.

**Figure 5:**
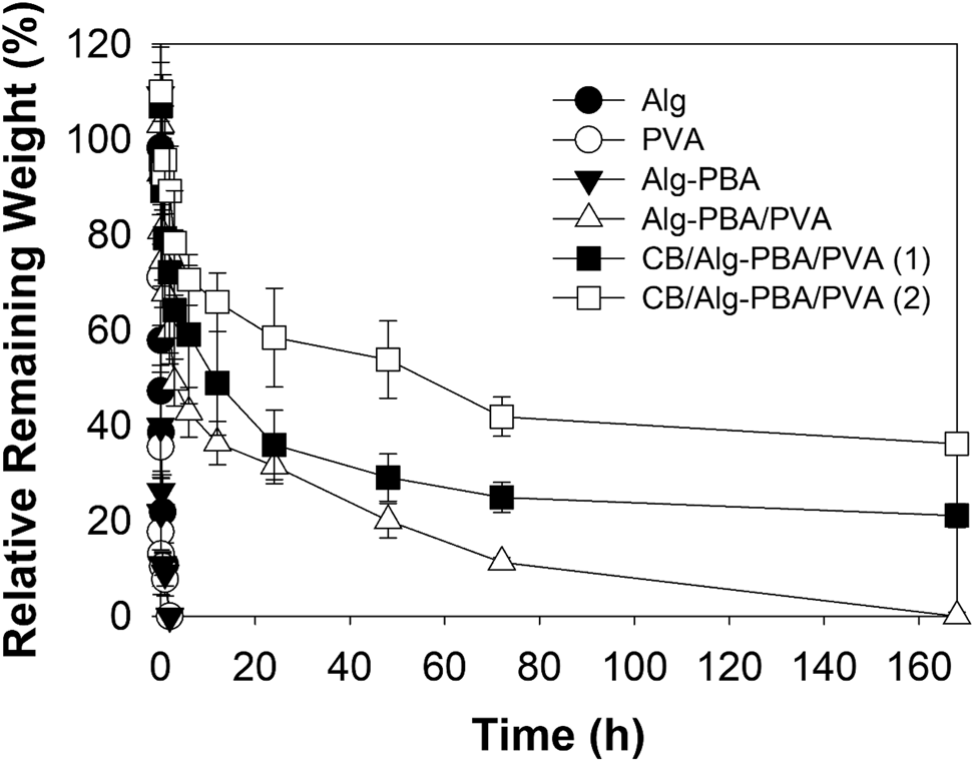
Relative remaining weight of Alg, PVA, Alg-PBA, Alg-PBA/PVA hydrogels, and CB/Alg-PBA/PVA hydrogels containing 1 or 2 mg CB at a predetermined time interval.

A significant property of endoscopic inks is tissue adhesiveness to the inner intestine, which prevents the malposition of tattooing inks. Alg-PBA alone shows excellent tissue adhesion to the exterior surfaces of the intestine^32^. The CB/Alg-PBA/PVA hydrogels also showed enhanced tissue adhesive properties compared with those of CB alone. We measured the adhesion forces using a lap shear test to evaluate the tissue adhesiveness of the hydrogels. As shown in Fig. 4a, the hydrogels were applied between the inners of the porcine intestines, and two tissues were attached to the hydrogels (Fig. 4a, left). Detachment forces were recorded by pulling the probe (Fig. 4a, right). The measured detachment stress of CB/Alg-PBA/PVA hydrogels containing 2 mg CB was 16.7 ± 0.5 kPa, higher than that of the CB alone (0.4 ± 0.1 kPa), as shown in Fig. 4b. In addition, the tissue adhesiveness of CB/Alg-PBA/PVA hydrogels were increased as a function of CB concentration (2.7 ± 0.9 kPa for 0 mg, 6.1 ± 1.6 kPa for 0.5 mg, 9.6 ± 2.0 kPa for 1 mg, and 16.7 ± 0.5 kPa for 2 mg CB). The adhesion forces of the hydrogels were remarkable with that of Alg-PBA hydrogels with ~ 50% of PBA substitution (13.9 ± 4.4 kPa)^32^. Furthermore, the CB/Alg-PBA/PVA hydrogels were long-lasting in pH 7.4 PBS solution compared with other compositions (i.e., PVA, Alg-PBA, Alg/PVA, and Alg-PVA/PVA). The remaining weights of the CB/Alg-PBA/PVA hydrogels were 36.1 ± 2.5% for 2 mg CB and 21.0 ± 2.5% for 1 mg CB, whereas Alg-PBA/PVA hydrogels disappeared within 160 h of incubation (Fig. 5). Also, elastic modulus values of CB/Alg-PBA/PVA hydrogels with 2 mg CB were 1.8 kPa after the stability tests for 160 h (Fig. S3). In addition, no Alg, PVA, and Alg-PBA were found after 2 h of incubation (Fig. S4). The results clearly show that CB/Alg-PBA/PVA hydrogels are potentially applicable as endoscopic inks owing to their superior self-healing, adhesive properties, and stability.

### Immobilizations of CB/Alg-PBA/PVA hydrogels on the tissue surfaces

**Figure 6:**
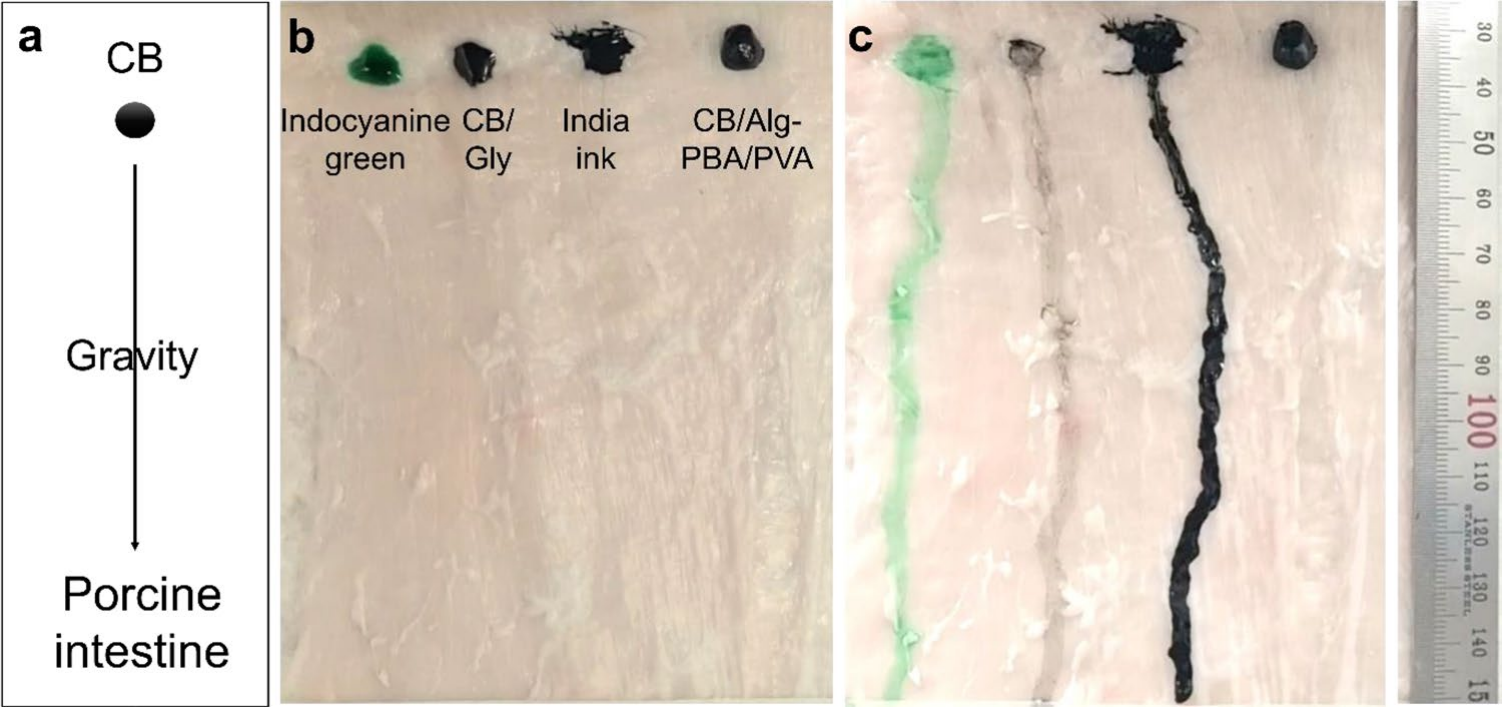
(**a**) Schematic illustrations of immobilization experiments of tattooing materials on porcine intestine. (**b,c**) photographic images of indocyanine green, CB, India ink, and CB/Alg-PBA/PVA hydrogels (**b**) before and (**c**) after experiments.

**Figure 7:**
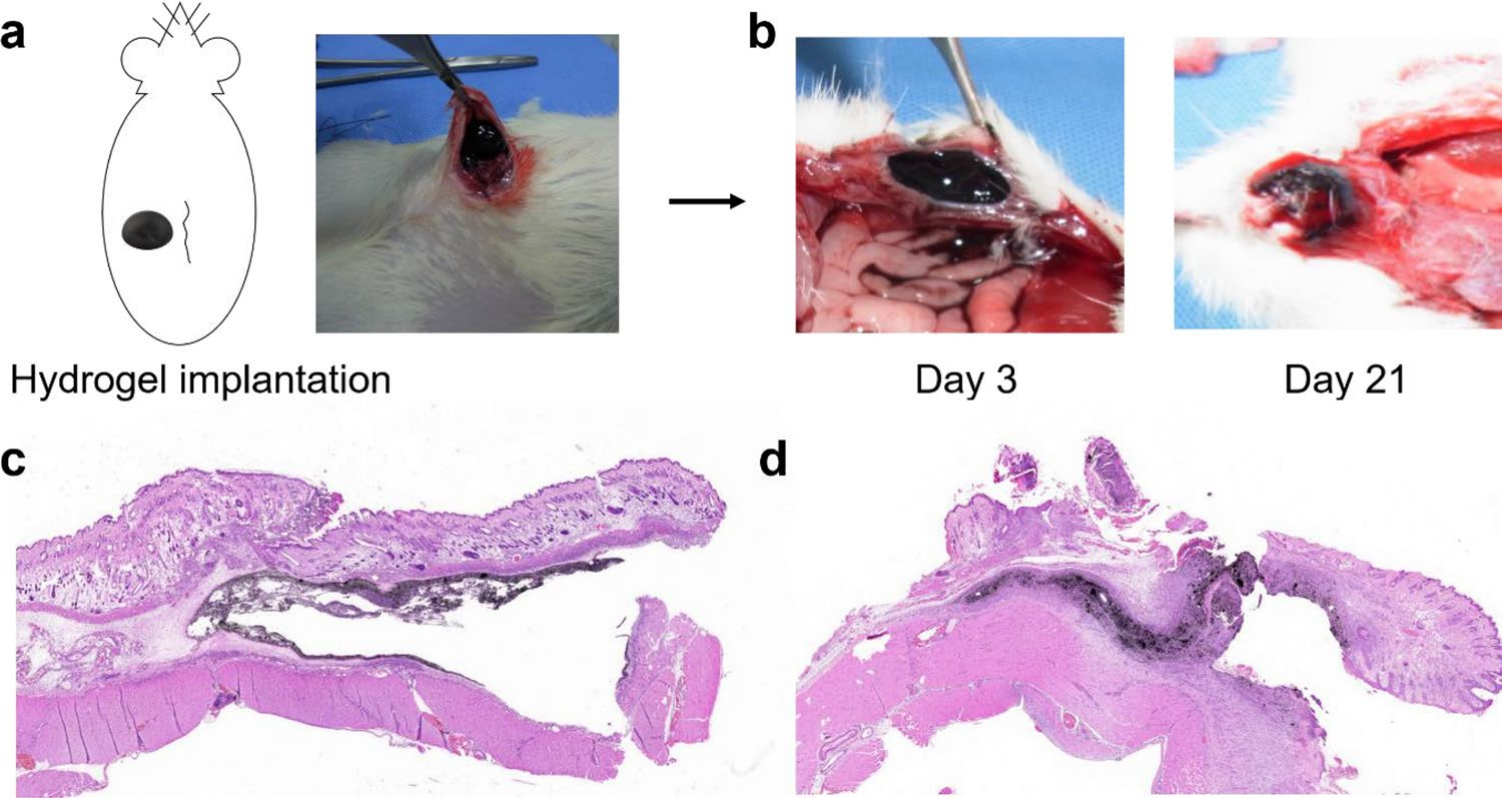
In vivo rat experiments for hydrogel implantations. (**a**) Experimental procedures for hydrogel implantations. (**b**) Photos of hydrogels on the rat abdominal wall after 3 (left) and 21 days (right). (**c,d**) Histological analysis of H/E stained rat intestine after 7 (**c**) and 14 days (**d**).

CB/Alg-PBA/PVA hydrogels were further tested for their potential application in endoscopic tattooing. As shown in Fig. 6a, the CB/Alg-PBA/PVA hydrogel was placed on the inner surface of the porcine intestine. The porcine intestine was tilted at an angle of approximately 90°, with the intestines maintained during the experiments. Figure 6b and c show the immobilization efficiency of endoscopic tattooing materials on the intestine. Before standing, no significant differences were observed between the samples (Fig. 6b). However, commercially available indocyanine green tattooing materials, CB in water and glycerin, and India ink flowed down immediately after standing (Fig. 6c). In contrast, the CB/Alg-PBA/PVA hydrogels adhered to the tissue surfaces and showed no flow during the experiments because of the synergistic effects of the tissue adhesiveness and mechanical properties. Interestingly, CB/Alg-PBA showed a flow, but most remained in the target tissues (Fig. S5a,b, left). Considering the usability of endoscopic inks, the CB/Alg-PBA solution may also be useful for surgical procedures. Next, we performed in vivo animal experiments to monitor the visibility of hydrogels as a function of time. As presented in the schematic diagram, the hydrogels were injected into the rat (Fig. 7a), and the visibility of the hydrogels was monitored at predetermined time intervals (3, 7, 14 and 21 days). A comparison of the amount after 3 and 21 days showed that the hydrogels were stably adhered to abdominal walls of the rat and were visible for at least 21 days (Fig. 7b). The postoperative area was identified, and the amount decreased and decomposed significantly. In addition, histological analysis supported the tattooing properties of the materials in the intestine (Fig. 7c,d). After performing histological analysis of H/E staining, the abdominal subcutaneous tissue of rats after 7 days revealed a marked amount of residual carbon tattoo particles with mild acute and chronic inflammation (Fig. 7c); however, after 14 days, moderate amounts of residual carbon tattoo particles with moderate acute and chronic inflammation were observed (Fig. 7d). Our findings suggest that self-healing adhesive polymeric hydrogels contribute to the improved immobilization and visibility of CB on the target tissues even though mild to moderate inflammatory responses were observed on days 7 and 14. Further study is warranted to reduce the tissue responses and enhance the accurate localizations of the potential lesions according to the decrease of polymer concentrations.

## Conclusion

In summary, we demonstrated self-healing adhesive CB/Alg-PBA/PVA hydrogels for use in endoscopic tattooing. The hydrogels exhibited self-healing, extensible, and re-shapable properties with tissue adhesiveness via phenylboronic acid-diol interactions. In addition, incorporating CB into the polymeric gel networks increased the elastic modulus values of hydrogels. Furthermore, CB/Alg-PBA/PVA hydrogels were successfully immobilized on the inner surface of the intestine with no flow against gravity. In addition, the hydrogels stably adhered to the peritoneum with visibility for at least 21 days in vivo. Thus, CB/Alg-PBA/PVA hydrogels have enormous potential for applications in various surgical situations, including endoscopic tattooing.

## Supplementary Information

Supplementary Figures.

## Data Availability

The data are available from the corresponding author on reasonable request.
