# Supplementary material for: Carbon black-containing self-healing adhesive hydrogels for endoscopic tattooing
Source: Sci Rep. 2023 Feb 2;13:1880. doi: 10.1038/s41598-023-28113-1 (PMC9895047; doi:10.1038/s41598-023-28113-1)
Supplement: Supplementary file 1 — Supplementary Figures. [file 41598_2023_28113_MOESM1_ESM.docx]

**Supplementary Information**

**Carbon black-containing self-healing adhesive hydrogels for endoscopic tattooing**

Hyung Jun Kwon^1,†^, Hyun Ho Shin^2,†^, Da Han Hyun^3^, Ghilsuk Yoon^4^, Jun Seok Park^1,*^, Ji Hyun Ryu^5,6,*^

^1^Department of Surgery, Kyungpook National University Hospital, School of Medicine, Kyungpook National University, Daegu 41404, South Korea

^2^Department of Chemical Engineering, Wonkwang University, Iksan, Jeonbuk 54538, South Korea

^3^Department of Biomedical Science, School of Medicine, Kyungpook National University, Daegu 41404, South Korea

^4^Department of Pathology, School of Medicine, Kyungpook National University, Daegu 41566, South Korea

^5^Department of Carbon Convergence Engineering, Wonkwang University, Iksan, Jeonbuk 54538, South Korea

^6^ICT Fusion Green Energy Research Institute, Wonkwang University, Iksan, Jeonbuk 54538, South Korea

^†^These authors contributed equally to this work

*Corresponding author

Prof. J. H. Ryu, E-mail: jhryu4816@wku.ac.kr

Prof. J. S. Park, E-mail: parkjs0802@knu.ac.kr


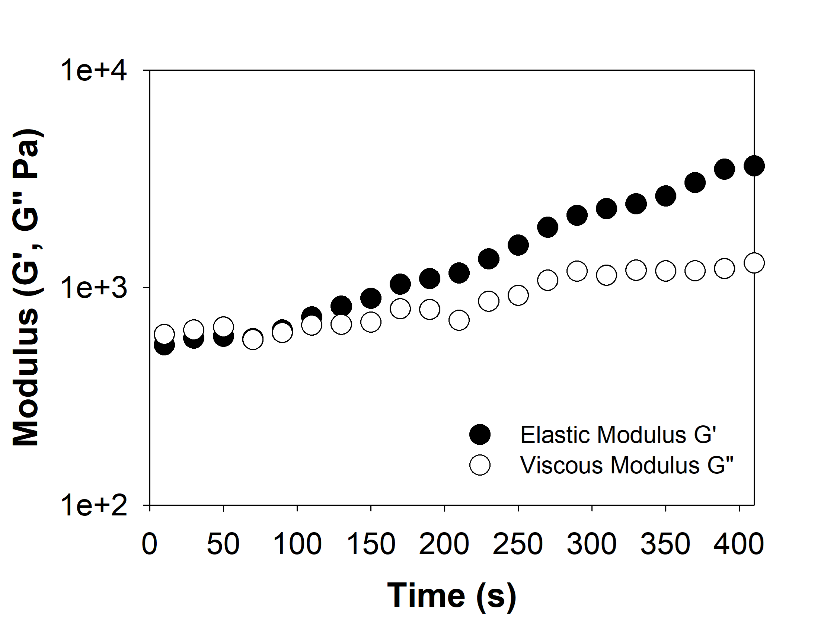


**Figure S1**. Gelation kinetics of CB/Alg-PBA/PVA hydrogels.


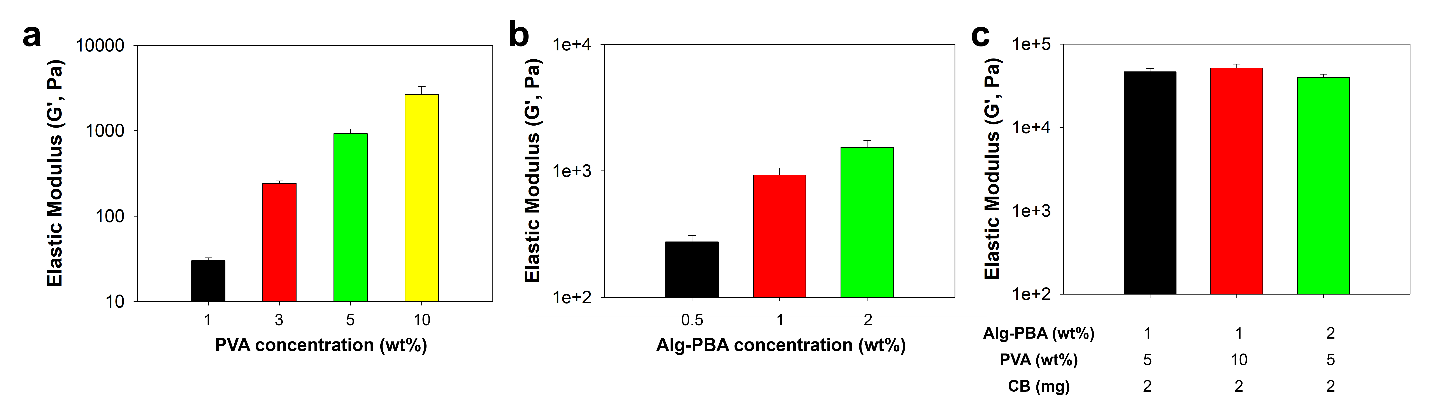


**Figure S2**. **a**) Elastic modulus values of Alg-PBA/PVA hydrogels as a function of PVA concentrations at the Alg-PBA concentration of 1 wt%. **b**) Elastic modulus values of Alg-PBA/PVA hydrogels at different concentrations of Alg-PBA polymer (PVA concentration: 5 wt%). **c**) Elastic modulus changes of CB/Alg-PBA/PVA hydrogels after addition of CB (2 mg).


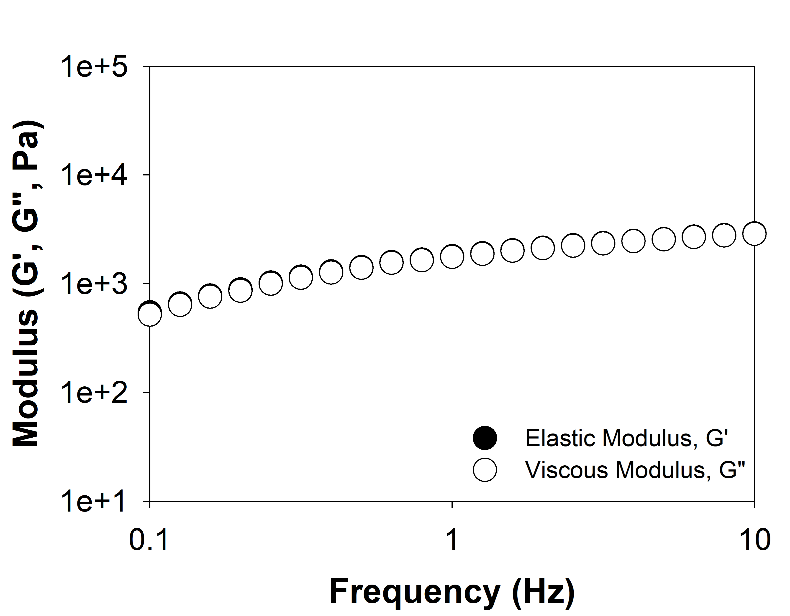


**Figure S3**. Frequency sweep measurements of CB/Alg-PBA/PVA hydrogels after 160 h of incubation.


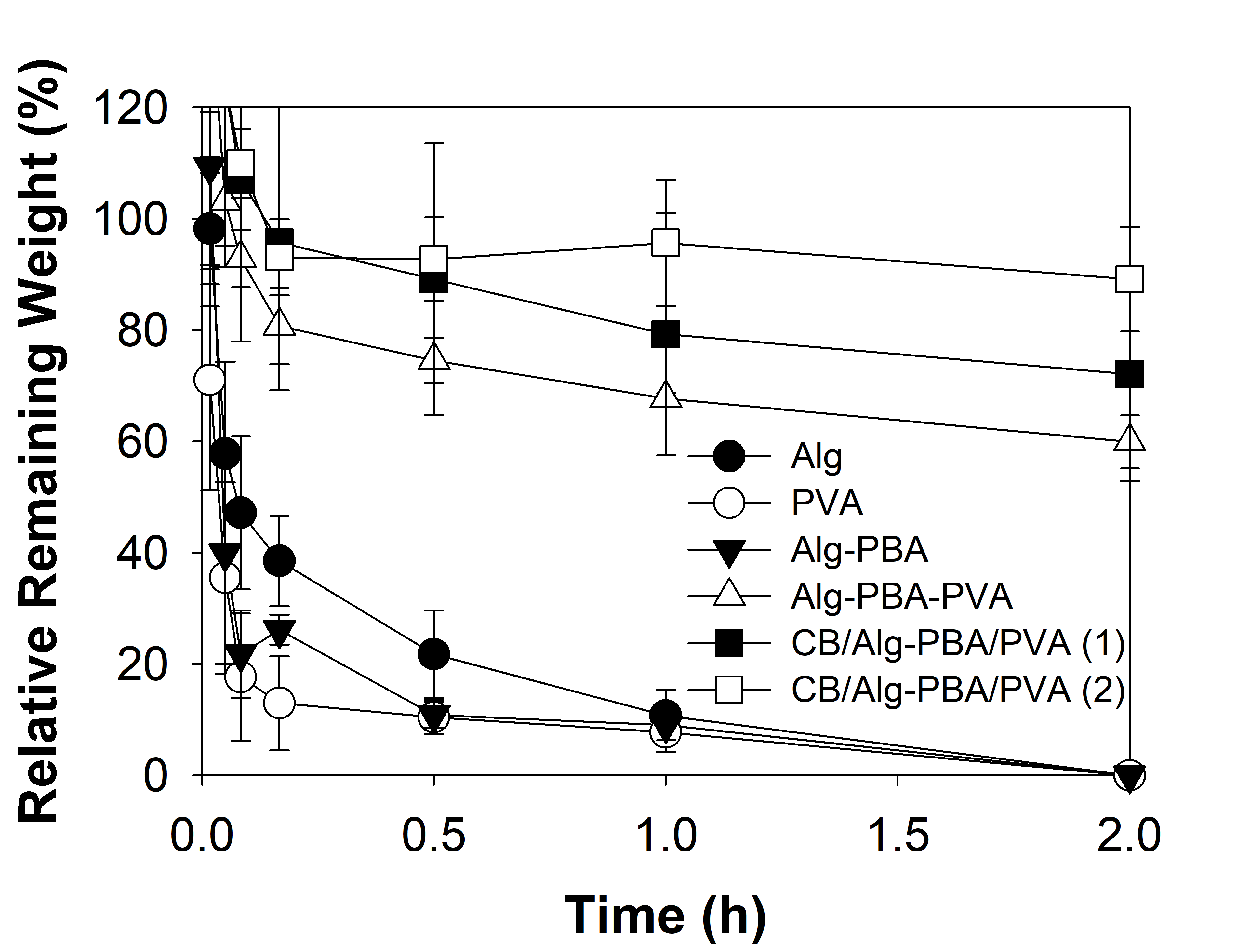


**Figure S4**. Relative remaining weight of Alg, PVA, Alg-PBA, Alg-PBA/PVA hydrogels, and CB/Alg-PBA/PVA hydrogels containing 1 or 2 mg CB until 2 h of incubation.


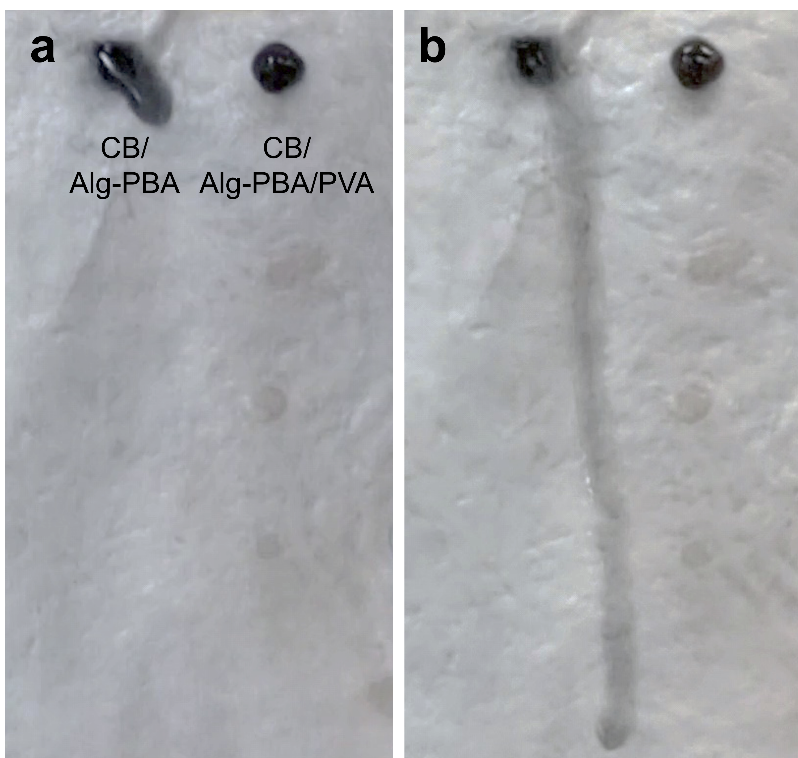


**Figure S5**. Photographic images of CB/Alg-PBA and CB/Alg-PBA/PVA hydrogels (**a**) before and (**b**) after CB-immobilization experiments on porcine intestine.
